# Low Doses of Kretek Cigarette Smoke Altered Rat Lung Histometric, and Overexpression of the p53 Gene

**DOI:** 10.2174/0118743064285619240327055359

**Published:** 2024-04-26

**Authors:** Edy Parwanto, David Tjahyadi, Sisca Sisca, Husnun Amalia, Nany Hairunisa, Hosea Jaya Edy, Ashaolu Victoria Oladimeji, Noureddine Djebli

**Affiliations:** 1 Department of Biology, Faculty of Medicine, Universitas Trisakti, Jl. Kyai Tapa, Kampus B, No.260 Grogol 11440, Jakarta, Indonesia; 2 Department of Histology, Faculty of Medicine, Universitas Trisakti, Jakarta, Indonesia; 3 Department of Ophthalmology, Faculty of Medicine, Universitas Trisakti, Jakarta, Indonesia; 4 Department of Occupational Medicine, Faculty of Medicine,Universitas Trisakti, Jakarta, Indonesia; 5 Study Program of Pharmacy, Faculty of Math, and Natural Sciences, Universitas Sam Ratulangi, Manado, Indonesia; 6 Department of Chemistry, Loyola Institute of Frontier Energy, Loyola College, Chennai, India; 7 Department of Biologie, Faculty of Natural and Life Sciences, Abdelhamid Ibn Badis University, Mostaganem, Algeria

**Keywords:** Filtered kretek cigarettes smoke, Histometric, Bronchioles, Respiratory bronchioles, p53 gene, Tobacco

## Abstract

**Background:**

The components of kretek cigarettes include tobacco as the main part, clove, and sauce. Filtered kretek cigarettes are kretek cigarettes that have one end filtered. Cigarette smoke contributes to the disruption of the respiratory system, so it is necessary to know the effect of low doses of cigarette smoke on changes in the histometric of the respiratory system, and whether it affects p53 gene expression. This study aims to determine changes in the histometric of the respiratory system and p53 gene expression.

**Methods:**

In this study, we used Sprague-Dawley rats. Group I of rats breathing normal air, were not exposed to filtered kretek cigarette smoke (as a control). Group II of rats, as a treatment group, were exposed to filtered kretek cigarette smoke 1 stick/day for 3 months. The results of lung histometry measurements and p53 gene expression between groups were analyzed using the Independent Sample T-test. The difference between groups is significant if the test results show P < 0.05.

**Results:**

Bronchioles length, width, area, and perimeter in group I were 40.55±1.57 μm, 14.82±0.41 μm, 494.61±5.62 μm^2^, and 233.87±4.51 μm, respectively. Bronchioles length, width, area, and perimeter in group II were 30.76±0.78 μm, 9.28±0.40 μm, 297.32±2.53 μm^2^, and 177.84±5.15 μm, respectively. The area and perimeter of respiratory bronchioles in group I were 17.68±0.49 μm^2^, and 26.60±0.52 μm respectively, while those in group II were 19.28±0.35 μm^2^, and 29.28±0.35 μm, respectively. Mucus was found in the bronchioles and respiratory bronchioles in group II, however, there was no visible mucus observed in group I. In addition, it was also concluded that exposure to low doses of filtered kretek cigarette smoke, 1 cigarette/day for 3 months, increased the expression of the p53 gene in the lungs of rats.

**Conclusion:**

The size of bronchioles in rats decreased after being exposed to filtered kretek cigarette smoke 1 stick/day for 3 months, while the size of respiratory bronchioles increased. In addition, exposure to filtered kretek cigarette smoke increased the expression of the p53 gene in the rat lungs.

## 1 INTRODUCTION

In Indonesia, many people smoke, especially among young people [1]. Cigarettes can be obtained from the market, but some are made by residents, manually. The main ingredient of cigarettes is tobacco. Cigarettes are one of the major health problems in the world. Even so, many people smoke. It has been reported that cigarettes kill more than 8 million people each year in the world. More than 7 million of those deaths are the result of direct tobacco use, while around 1.2 million are the result of non-smokers being exposed to second-hand smoke [2]. The other reports demonstrated that smoking is a risk factor for various types of diseases, because smokers should stop smoking before the age of 40 years [3]. Especially Indonesia, is one of the countries in Southeast Asia whose population is mostly smokers [4]. Moreover, the number of smokers in Indonesia is very large. In addition, filtered kretek cigarettes are in great demand by most active smokers in Indonesia. This fact shows that most Indonesians smoke kretek.

There are several types of cigarettes in Indonesia, including filtered kretek cigarettes. Generally, filtered kretek cigarettes are made in the factory using machines and are equipped with a filter at one end. In an earlier period, the kretek cigarettes were not filtered at one end. Until now, on the market circulating filtered kretek cigarettes, and non-filtered kretek cigarettes. Filtered kretek cigarettes contain tobacco (as the main ingredient) that is mixed with cloves and sauces [5]. In addition to the added cloves, there are other ingredients to complement the taste of kretek cigarettes, namely sauce.

The scientific name of cultivated tobacco is *Nicotiana tabacum* Linn. Based on a previous report, the Indonesia cigarette industry (±80%) used local tobacco such as Temanggung tobacco, Weleri tobacco, Yogyakarta tobacco, Muntilan Tobacco, Boyolali tobacco, Paiton tobacco, Kasturi tobacco, and Madura tobacco [6]. The dried clove flower buds (*Syzygium aromaticum* (L.) Merr. & L.M.Perry) [7] were used as dough in filtered kretek cigarettes. Filtered kretek cigarette sauce is a unique taste of cigarettes [8]. In Klaten district and its surroundings (Central Java Province, Indonesia), kretek cigarette sauce is known as “woor”.

Cigarette smoke contains a complex mixture of chemical compounds. More than 4000 chemical compounds in cigarette smoke have been identified [9]. Cigarette smoke is known to consist of gas (92%) and solid or particulate matter (8%) [10]. Recent research demonstrates that particulate matter emissions from kretek cigarettes can be very high. This is clearly dangerous for both active smokers and non-smokers who are exposed to cigarette smoke [11]. People who smoke, gases and particulate matter in the smoke pour from the mouth to the alveoli. It has been demonstrated that there was deposition of cigarette smoke particles in various locations of the human respiratory tract [12].

Furthermore, it has been demonstrated that cigarette smoke is associated with bronchopulmonary dysplasia [13]. In addition, cigarette smoke also has free radicals that cause airway disorders, resulting in lung abnormalities. Moreover, cigarette smoke contains particles that damage lung tissue, causing permanent damage called chronic obstructive pulmonary disease (COPD) [14, 15].

Generally, a rat’s respiratory system consists of the nose, pharynx, larynx, trachea, primary bronchi, and lungs (bronchioles and alveoli) [16]. The latest research demonstrated that components of rat lung structure are blood vessels, bronchi, terminal bronchi, respiratory bronchioles, alveolar ducts, alveolar sacs, and alveoli [17]. In addition, it has been demonstrated about the lower respiratory system of mice (bronchioles, alveolar passages, and alveoli) both qualitatively and quantitatively [18]. A previous study reported that the respiratory systems of rats exposed to gas showed increased tissue elasticity and tissue resistance [19]. A recent study also reported that rats were given conventional cigarette smoke, collagen deposition appearance in the bronchioles, as well as intrabronchiolar mucus [20].

In addition, it is also shown that cigarette smoke affects the anatomical structures of the respiratory system [21]. Another study also showed that smokers have experienced basal cell hyperplasia, mucous cell hyperplasia, and squamous metaplasia [22]. In the application of COPD diagnosis, both qualitative and quantitative assessments are needed [23]. This research is important because bronchioles and respiratory bronchioles are an important part of the lungs associated with the alveoli. We intend to present the quantitative changes in bronchioles, and respiratory bronchioles caused by the effects of filtered kretek cigarette smoke. Quantitative changes in the bronchioles and respiratory bronchioles are important so that the diagnosis related to lung tissue disorders can be determined more objectively. Apart from that, it also needs to be proven whether low doses of kretek cigarette smoke affect gene expression, especially p53. We know that the p53 gene is used as a tumour marker as a key cellular process such as proliferation, apoptosis, and metabolism to suppress tumorigenesis [24]. Therefore, based on the description above, it is necessary to research the histometric of bronchioles, and respiratory bronchioles in Sprague-Dawley rats after treatment with filtered kretek cigarette smoke. Likewise, it is also necessary to prove the dangers of low-dose smoking on over-expression of the p53 gene.

## 2 MATERIALS AND METHODS

### 2.1 Preparation of Filtered Kretek Cigarettes

The type of tobacco used as an ingredient in filtered kretek cigarettes was local tobacco. The types of local tobacco (*Nicotiana tabacum* Linn.) were collected from areas in Manisrenggo District, Klaten Regency, Central Java Province, Indonesia. Cloves (*Syzygium aromaticum* Merr. & L.M. Perry) used in filtered kretek cigarette dough were dried clove flowers, collected from areas in the Logede Village, Karangnongko District, Klaten Regency, Central Java Province, Indonesia. The sauce known as “woor,” used for filtered kretek cigarettes was a special ingredient that was always used to mix cigarettes manually. These materials were used to make filtered kretek cigarettes manually.

### 2.2 Ethical Clearance

The ethical clearance of this research was obtained from the Research Ethics Commission, Faculty of Medicine, Universitas Trisakti, Jakarta, Indonesia (No. 184/KER-FK/VIII/2018).

### 2.3 Flowchart of Experimental Rat Treatment

The flowchart of experimental rat treatment is presented in Fig. (**1**).

### 2.4 Animal Care

We used *Rattus norvegicus*, Sprague-Dawley strain. The number of rats per group was calculated using formula (n-1) (t-1) ≥ 15. For each treatment group a minimum of 8 rats were required. Sixty male rats were used in this study. The rats included in this study were 2-3 months old and weighed 150-200 grams. Determination of rat health was carried out after examination by a Veterinary. Rats were caged (individually) in an air-conditioned room and the room temperature was set to 22±3 °C, humidity 55±5%, and artificial fluorescent lamps (12:12 hour light and dark cycle). The rats fed and drank in the libitum according to the standard. There are 2 stages in this research, namely the 1^st^ stage in the form of rat acclimatization and the 2^nd^ stage was the treatment of giving filtered kretek cigarette smoke to rats.

#### 2.4.1 Purchasing Details of the Experimental Rats

Type of animal is rat, Amount 16, Gender is Male Age is 2.5 months Weight 150-200 grams. Systematica (American Fancy Rat and Mouse Association, 2004) - Kingdom - Filum - Sub Filum - Class - Ordo - Sub Ordo - Family - Genus - Species - Strain: Animalia: Chordata: Vertebrata: Mammalia: Rodentia: Myomorpha: Muridae: Rattus: Rattus novergicus: Sprague Dawley

### 2.5 Treatment on Sprague-dawley Rats

Trial of filtered kretek cigarette smoke on rats was carried out at The Veterinary Medical Teaching Hospital (Rumah Sakit Hewan Pendidikan=RSHP), Faculty of Veterinary Medicine of Bogor Agricultural University. This research was conducted between November 2019 - April 2020.

**Fig. (2):**
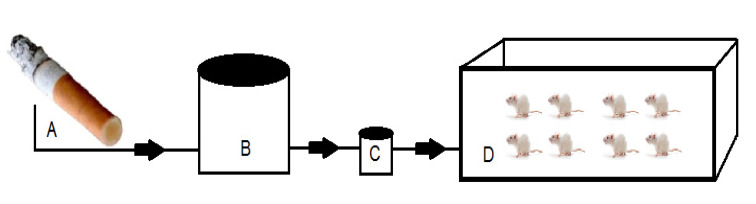
Schematic diagram of filtered kretek cigarettes smoke exposure to a groups of Sprague-Dawley rats. **A**. Filtered kretek cigarettes burned. **B**. Tube to accommodate filtered kretek cigarettes smoke. **C**. Electric pump. **D**. Glass box for rats treatment with length 40 cm, width 30 cm, and height 20 cm).

Acclimatization of rats was carried out for 2 weeks, then continued with treatment according to groups. Rats were divided into 2 groups with randomization. Group I was the rat group that was breathing normal air, and was not exposed to filtered kretek cigarette smoke (as a control). Group II, namely the rat group exposed to filtered kretek cigarettes smoke 1 stick/day/group (Fig. **2**). The treatment duration for the two groups of rats was 3 months. After the treatment, the rats were sacrificed, and then their respiratory organs were collected from the trachea to the lungs to make histology slides.

### 2.6 Measurement of Bronchioles and Respiratory Bronchioles

The tissue was immersed in a neutral buffer solution of 10% formalin for 24 hours at room temperature. The tissue was cut to a size of 1×1×1 cm, and then put in a tissue cassette. The tissue was then transferred to alcohol dehydration with alcohol concentrations of 70%, 80%, 90%, and 96%, respectively. The dehydration time was 2 hours for each alcohol concentration. The next stage was clearing, after which, the tissue was ready to be inserted into the paraffin block. Furthermore, embedding and blocking were carried out. The tissue in the paraffin block was cut using a microtome with a thickness of 4-5 μ, and then stained with hematoxylin and eosin.

Image documentation using Optilab Advance Plus and Image Raster 3 by PT MICONOS, Daerah Istimewa Yogyakarta, Indonesia; available at https://miconos.ac.id/new/support/download. Optilab Advance Plus and Image Raster 3 programs were used to analyze rat respiratory system histometrics (bronchioles and respiratory bronchioles) by three observers. Data for all groups were expressed as mean± standard deviation. Analyzing data between groups using an independent sample t-test. The difference between groups was significant if the test results showed P<0.05.

### 2.7 Expression of p53 Gene in Rat Lung

Measurement of p53 gene expression was carried out using real-time polymerase chain reaction (RT PCR). Lung tissue samples of Sprague Dawley rats were isolated for mRNA, and then the solution was put into a 1.5 mL tube, stored at –80 ^o^C. The reagents for measuring mRNA levels were Bioline SensiFast Sybr Lo-ROX One-step kit, Zymo Research Quick-RNATM MiniPrep Plus, and Geneaid Micropestle. The p53 rat target gene used the sense primer (5' to 3') AGGCCTTGGAACTCAAGGAT, while the antisense primer (5' to 3') TGAGTCAGGCCCTTCTGTCT, witha sizeof 140bp. Theglyceraldehyde-3-phosphate dehydrogenase (GAPDH) gene was used as an internal control. The sense primer of GAPDH gene was (5' to 3') CCAGGTGGTCTCCTCTGACTTCTC, while the antisense primer was (5' to 3') ATACCAGGAAATGAGCTTGACA, with a size of 147 bp [25]. RT PCR analysis using SYBR Green Master Mix (Bio-rad) in the Bio-rad CFX96 RT PCR system. Ct p53 gene was normalized with Ct gene GAPDH. The calculation was based on the 2^-∆∆C^T method (Livak method) [26], as demonstrated in Table **1**.

## 3 RESULTS

### 3.1 Filtered Kretek Cigarettes

The constituent materials of filtered kretek cigarettes are presented in Fig. (**3**). Filtered kretek cigarettes consist of filters and kretek dough. Tobacco is the main ingredient of kretek cigarettes, which is mixed with cloves and sauce.

### 3.2 Hair Appearance

The hair appearance of rats between group I and group II is presented in Fig. (**4**). The hair on the back and abdomen of rats in group I looks white, clean, and dense, while in group II, it looks dull yellow, less clean, and sparse.

### 3.3 Bronchioles Histometric

Histological of bronchioles in Sprague-Dawley rats between group I and group II is presented in Fig. (**5**). Treatment of filtered kretek cigarettes smoke 1 stick/day for 3 months causes mucus in the bronchioles space of rats in group II, while in group I, it is not found. In addition, the size of the bronchioles space also appears to be shrinking.

A comparison of bronchioles histometric in Sprague-Dawley rats between group I and group II is presented in Fig. (**6**). Based on Fig. (**6**), seen that bronchioles length, width, area, and perimeter in group I are different compared to group II (P=0.000). Bronchioles histometric of rats in group II<I (P=0.000). In this study, the group of rats exposed to filtered kretek cigarette smoke had changes in bronchioles histometric, namely the decrease in length, width, area, and perimeter. The bronchioles length of rats in group II decreased by 24.14% compared to group I, while the bronchioles width of rats in group II decreased by 37.38% compared to group I. The bronchioles area of rats in group II decreased by 39.89% compared to group I, while the bronchioles perimeter of rats in group II decreased by 23.96% compared to group I. Rats in group II exert a negative response against filtered kretek cigarette smoke in the form of a decrease in the size of bronchioles histometric.

### 3.4 Respiratory Bronchioles Histometric

The histological of respiratory bronchioles in Sprague-Dawley rats between group I and group II is presented in Fig. (**7**). Based on Fig. (**7**) treatment of filtered kretek cigarettes smoke 1 stick/day for 3 months causes mucus in the respiratory bronchioles space of Sprague-Dawley rats in group II, while in group I is not found.

The area and the perimeter of respiratory bronchioles in the Sprague-Dawley rats between group I and group II is presented in Fig. (**8**). Fig. (**8**) demonstratesed that the area and the perimeter of respiratory bronchioles of rats in group I < group II (P=0.000). In this study, the group of rats exposed to filtered kretek cigarette smoke had changes in respiratory bronchioles histometric, namely the increase in area and perimeter. The area of respiratory bronchioles of rats in group II increased by 9.05% compared to group I, while the perimeter of respiratory bronchioles of rats in group II increased by 10.075% compared to group I. Rats in group II had a positive response against filtered kretek cigarette smoke in the form of an increase in the area and perimeter of respiratory bronchioles.

### 3.5 Expression of p53 Gene

Optimization of RT PCR for the determination of mRNA levels for p53 is presented in Fig. (**9**). Based on the measurement data, it was demonstrated that the treatment of rats exposed to filtered kretek cigarette smoke 1 cigarette/day for 3 months influenced p53 gene expression. There was an increase in p53 gene expression in rats exposed to filtered kretek cigarette smoke. It was proven that the mRNA levels of group II were higher (upregulation) than group I (P=0.000).

## 4 DISCUSSION

The color of the filter on filtered kretek cigarettes before burning was different from after burning. Before the filtered kretek cigarettes were burned, the filter waswhite, while after they were burned, the filter had a burlywood color. It has been demonstrated that the function of the filter at the end of the filtered kretek cigarettes is to reduce tar and nicotine levels that enter the respiratory tract. The color change of the burlywood filter in this study indicated the presence of tar and nicotine from filtered kretek cigarettes. This fact is in line with previous research [27].

Filtered kretek cigarettes that were burning produced cigarette smoke and ash. Cigarette smoke was inhaled by rats, while ash of filtered kretek cigarettes was disposed of. Most of the filtered kretek cigarette smoke entered the respiratory system of rats. Filtered kretek cigarette smoke is one of the incomplete combustion products of tobacco, cloves, and sauce. Cigarette smoke includes main cigarette smoke and side cigarette smoke. Main cigarette smoke is cigarette smoke that was inhaled by rats as a model of active smokers. Side cigarette smoke is cigarette smoke that wasin the environment around rats. In this study, filtered kretek cigarettes burned, and a pump mainly inhaled cigarette smoke and then channeled into the smoking box. This is similar to the smoking process that is generally carried out by smokers. The results of this study clearly show that there are differences in the color of the filters before and after the filtered kretek cigarettes burned. This fact clarifies the filter function of the filtered kretek cigarettes, which is to filter particles in cigarette smoke that are inhaled by smokers. Therefore, the filteron filteredkretek cigarettescan reducethe content of substances inhaled when smoking. Substances in cigarette smoke that have been shown to be high in content include nicotine, various metals [28], free radicals [29], oxidants and antioxidants [30]. Even though there is a filter, cigarette smoke in this study was proven to affect the hair appearance of rats, which worsened hair appearance.

Exposure to filtered kretek cigarette smoke in this study caused the hair of rats to change from pure white to dull yellow. The hair discoloration occurred throughout the rat's body. The results in this study were in accordance with the results of the report which stated that the smoker's mustache hair turned yellow [31]. Filtered kretek cigarette smoke in this study mostly comes from tobacco leaves, so it has nicotine. Nicotine in the filtered kretek cigarette smoke enters the body and then causes discoloration, cleanliness, and density of hair in the rats. This fact is in accordance with the results of earlier studies, which show that the nicotine content of hair is used as a biomarker to assess chronic exposure to environmental tobacco smoke [32]. Nicotine in cigarette smoke by inhalation enters the body and penetrates cell membranes to the systemic circulation system. It is further stated that nicotine is the main constituent of tobacco. Nicotine is not carcinogenic, but it triggers many carcinogens in tobacco [33]. Previous research has demonstrated that nicotine levels in the hair of smokers are much higher compared to non-smokers [34]. In addition to nicotine content, cigarette smoke also causes oxidative stress that affects the condition of the hair and, among others, affects the hair fiber [35]. In addition to affecting hair color, cigarette smoke also affects histometrics in the lungs of rats, including bronchioles and respiratory bronchioles.

The reduction in length, width, area, and perimeter of bronchioles in rats in this study responded to unwanted cigarette smoke exposure because, in normal conditions, the respiratory air does not contain cigarette smoke. This occurs as a defense mechanism for reducing the volume of cigarette smoke exposure. Reduction of bronchioles histometric reduces exposure to filtered kretek cigarette smoke, next to reducing the volume of cigarette smoke until it reaches the alveolar sacs (alveolar saccules). This situation allows obstruction of airflow that cannot flow back completely. This fact is consistent with earlier studies showing that the bronchioles are the site of airway obstruction. In addition to bronchioles abnormalities due to cigarette smoke, it was found that cigarette smoke also causes COPD [36]. Based on the facts obtained, we agree that pulmonary histometrics is useful for quantitatively measuring the tissues that make up the organs in the respiratory system. Moreover, it has been demonstrated that exposure to filtered kretek cigarettes at a dose of 1 cigarette/day or 2 cigarettes/day for 30 days, causes bronchial mucosal hyperplasia and bronchoconstriction in male Sprague Dawley rats [37]. Apart from that, it has also been demonstrated that filtered kretek cigarette smoke not only impacts the respiratory organs but also impacts Purkinje cells and pyramidal cells in Sprague Dawley rats [38].

In addition, the facts in this study, in accordance with the results of other studies which, indicated that structural abnormalities and blockage by mucus can cause destabilization of the airways in the bronchioles [39]. Previous studies have shown that morphometric measurements of rat lungs have been carried out [17]. In addition to the morphometric analysis of the rat lungs, pulmonary histometrics have also been shown. The results of the study indicated that the diameter of the respiratory bronchioles, alveolar ducts, and alveoli were 24.93±1.27 µm, 21.14± 0.66 µm, and 12.95±0.21 µm, respectively [18]. Need to note that it is not only cigarette smoke that causes chronic obstruction of the bronchioles. It has been shown that chronic obstruction of the bronchioles can also be caused by cell degeneration in the bronchioles [40]. Therefore, it is still necessary to prove the effect of cigarette smoke on cell death in the bronchioles.

The effect of filtered kretek cigarette smoke in this study caused the area and perimeter of respiratory bronchioles in group II of rats to be greater compared to group I as a control. The effect of filtered kretek smoke in this study narrowed the bronchioles space (Fig. **6**) but widened the respiratory bronchioles space (Fig. **8**). The increased respiratory bronchioles space may be due to the pressure of filtered kretek cigarette smoke, such as the alveolar sac. The changes in histometric in the respiratory bronchioles in this study are in line with the other studies showing significant changes in the respiratory bronchioles of rats after treatment with hypoxia and melatonin [41].

**Fig. (5):**
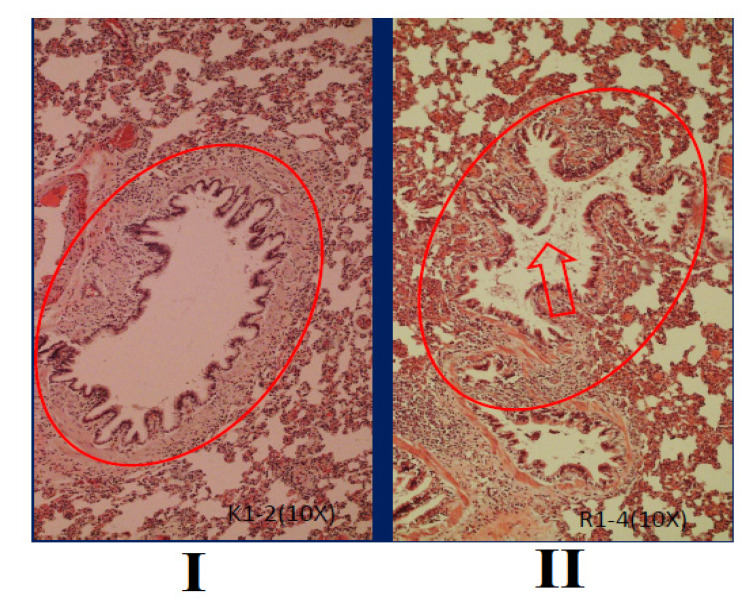
Bronchioles histological of Sprague-Dawley rats, stained with hematoxylin and eosin, objective10x. I. Group I, rats breathe using ordinary air without exposure to filtered kretek cigarette smoke. II. Group II, the group of rats exposed to filtered kretek cigarette smoke 1 stick/day for 3 months of treatment. The red arrows showed mucus.

**Fig. (6):**
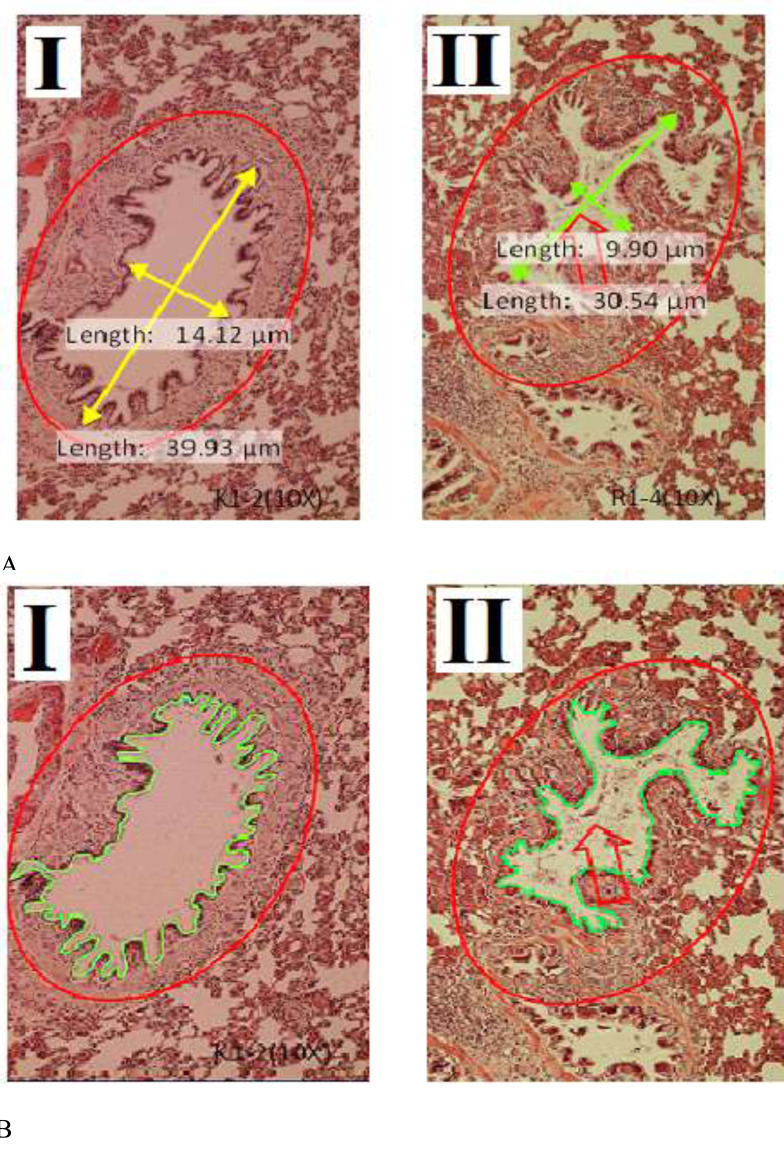
Comparison of bronchioles histometric in Sprague-Dawley rats between group I compared to group II, staining with hematoxylin and eosin, objective10x. (**A**). Length and width of the bronchioles. (**B**). Area and perimeter of the bronchioles. I. Group I, rats breathe using ordinary air without exposure to filtered kretek cigarette smoke. II. Group II, the group of rats exposed to filtered kretek cigarette smoke 1 stick/day for 3 months of treatment. The red arrows showed mucus.

**Fig. (7):**
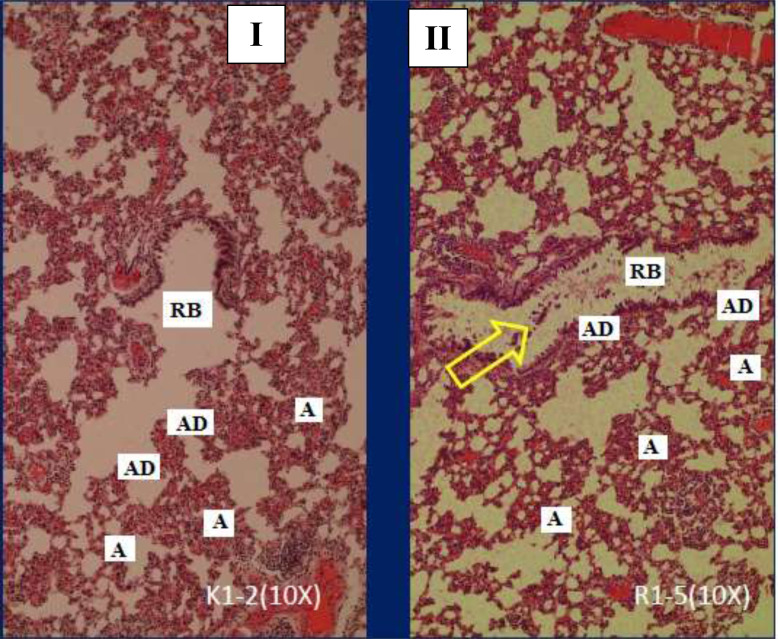
The histological of respiratory bronchioles in Sprague-Dawley rats between group I compared to group II, stained with haematoxylin and eosin, objective 10x. The yellow arrows showed mucus. RB= respiratory bronchioles, AD= alveolar ducts, A=alveoli. I. Group I, rats breathe using ordinary air without exposure to filtered kretek cigarette smoke. Group II, the group of rats exposed to filtered kretek cigarette smoke 1 stick/day for 3 months of treatment.

**Fig. (8):**
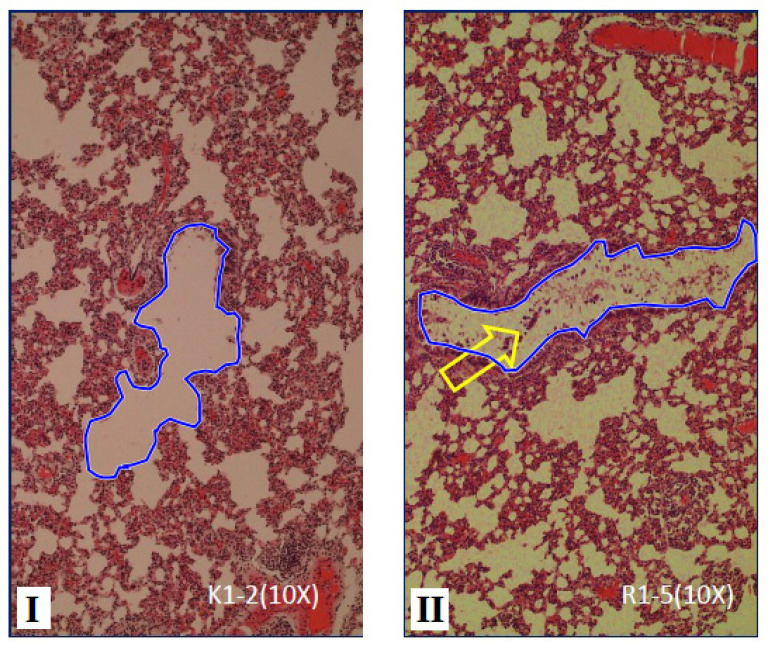
Comparison of area and perimeter of respiratory bronchioles in Sprague-Dawley rats between group I compared to group II, stained with haematoxylin and eosin, objective 10x. Respiratory bronchioles of rats in group II showed mucus (yellow arrow), while in the group I mucus was not found.I. Group I, rats breathe using ordinary air without exposure to filtered kretek cigarette smoke. II. Group II, the group of rats exposed to filtered kretek cigarette smoke 1 stick/day for 3 months of treatment.

Our results showed that mucus appeared in the bronchioles and respiratory bronchioles of rats. Filtered kertek cigarette smoke 1 cigarette/day for 3 months caused mucus in the respiratory bronchioles space in group II, while in group I (control), this mucus was not found (Figs. **5**-**8**). Of note, apart from in the bronchioles and respiratory bronchioles, mucus is also found in the saccus alveolar space (data not shown). The mucus is a complex mixture of mucins. Mucins are produced by airway epithelial cells and submucosal glands [42]. As a result of mucus that has accumulated in the bronchioles and respiratory bronchioles, airflow in the lungs is limited, thus resulting in lung disorders. In addition, demonstrated that inflammatory cells produce excess mucus which reduces lung function [43].

Previous studies demonstrated that cigarette smoke causes oxidative stress due to an imbalance between oxidants and antioxidants. Furthermore, cigarette smoke also induces epithelial cells for mucus hypersecretion, increasing proinflammatory cytokines and chemokines. Cigarette smoke has also been shown to increase macrophages and neutrophils and disrupt the balance of lymphocytes [30]. Cigarette smoke causes mucus hypersecretion and COPD. In addition, exposure to cigarette smoke can worsen COPD [44]. The results of other studies have also demonstrated that smoking at a young age is a dominant factor in the cause of COPD [45]. Based on the results of this study and several other studies, it is clear that cigarette smoke has a complex effect on lung tissue [46]. Moreover, cigarette smoke induces epigenetic changes in lung tissue [47], lung cancer [48, 49], oral disease, myocardial infarction, oral cancer, chronic health disease, cardiovascular disease, and asthma [50].

The Ct of the p53 gene for group I is 1, while the Ct p53 gene for group II shows a multiple of 1, so it experiences upregulation [51]. Of the 8 samples in this study, all showed up-regulation, and none showed downregulation. Rats exposed to filtered kretek cigarette smoke showed increased lung mRNA levels of the p53 gene. The occurrence of up regulation of the p53 gene in mice exposed to low doses of filtered kretek cigarette smoke in this study is in accordance with the statement relating to the processes of proliferation, apoptosis, and metabolism to suppress tumorigenesis [24]. It was further stated that the p53 gene plays an important role in responding to various types of stress cells [52]. The results of this study can be used as a basis for further research on tobacco consumption (cigarettes) with the occurrence of lung cancer. This is in accordance with research results, which demonstrate that tobacco use and variations in the p53 gene increase the risk of oral cancer [53, 54].

## 5 LIMITATIONS

The present study has limitations; among others, we did not measure the levels of chemical compounds in filtered kretek cigarette smoke. In this study, we have not determined damage to the bronchioles and respiratory bronchioles based on histometrics due to exposure to filtered kretek cigarette smoke. In addition, we have not performed cytometrics on bronchioles and respiratory bronchioles as we did before with human cervical cells [54, 55]. We hope that in the next opportunity, we can demonstrate the relationship between the levels of chemical compounds in filtered Kretek cigarette smoke with the level of the bronchioles and respiratory bronchioles damage based on cytometric and histometric.

## CONCLUSION

Based on the results obtained, we concluded that Sprague-Dawley rats in group II were exposed to 1 stick/day of filtered kretek cigarette smoke for 3 months and showed dull yellow hair on the back and abdomen, less clean, and less frequent compared to the group I (control). The size of bronchioles of Sprague-Dawley rats decreased after being exposed to filtered kretek cigarettes smoke 1 stick/day for 3 months, while the size of respiratory bronchioles increased. Mucus is found in the bronchioles and respiratory bronchioles of Sprague-Dawley rats. Mucus was found in the bronchioles, and respiratory bronchioles in the group were exposed to 1 stick/day of filtered kretek cigarette smoke for 3 months, while in the control group, there was no visible mucus. It is clear that the quantitative changes in bronchioles and respiratory bronchioles are caused by the effects of filtered kretek cigarettes smoke. Quantitative changes in the bronchioles and respiratory bronchioles are important so that the diagnosis related to lung tissue disorders can be determined more objectively. In addition, it was also shown that exposure to low doses of filtered kretek cigarette smoke, 1 cigarette/day for 3 months, increased the expression of the p53 gene (upregulation) in the rat lungs.

## SIGNIFICANCE STATEMENT

This study discovered that exposure to low-dose filtered kretek cigarette smoke changed the histometric of bronchioles and respiratory bronchioles in the Sprague-Dawley rats model. In addition, exposure to low-dose filtered kretek cigarette smoke also showed an increase in p53 gene expression in rat lungs.

## Figures and Tables

**Fig. (1):**
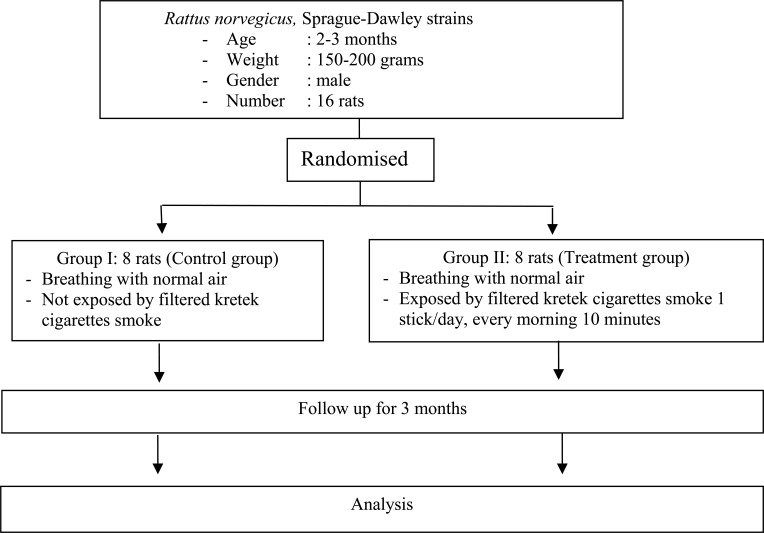
Flowchart of experimental rat treatment.

**Fig. (3):**
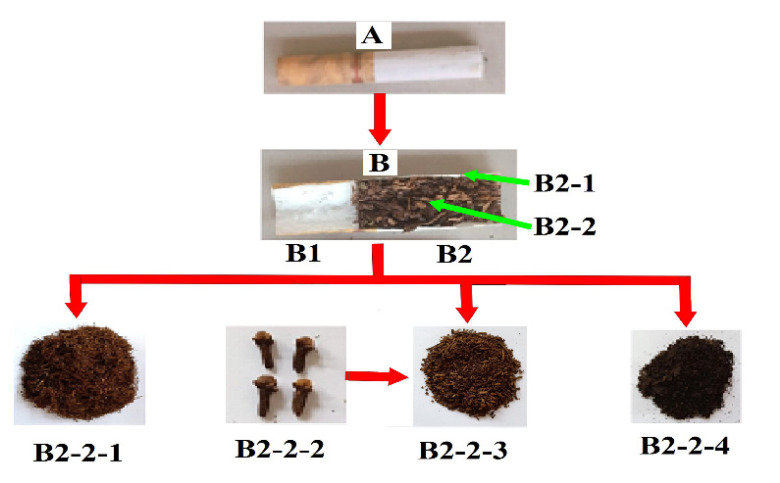
Composition of filtered kretek cigarettes. (**A**). The appearance of filtered kretek cigarettes. (**B**). Filtered kretek cigarettes split lengthwise so that it appears the inside which consists of the filter (B1) and kretek section (B2). B2-1. Wrapping paper for filtered kretek cigarettes dough. B2-2-1. Tobacco as the main constituent of filtered kretek cigarettes dough. B2-2-2. Dried clove flower. B2-2-3. Chopped dried clove flower. B2-2-4. Kretek sauce.

**Fig. (4):**
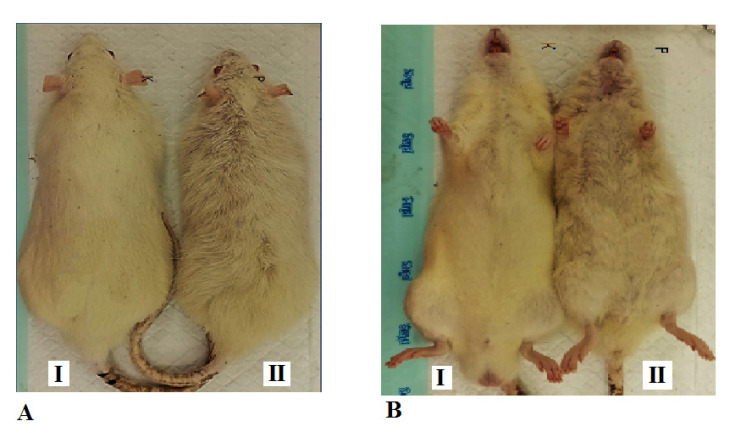
Hair of the Sprague-Dawley rats. **A**. Hair appearance of the back body. **B**. Hair appearance of the abdomen. I. Group I, rats breathe using ordinary air without exposure to filtered kretek cigarette smoke. II. Group II, the group of rats exposed to filtered kretek cigarette smoke 1 stick/day for 3 months of treatment.

**Fig. (9):**
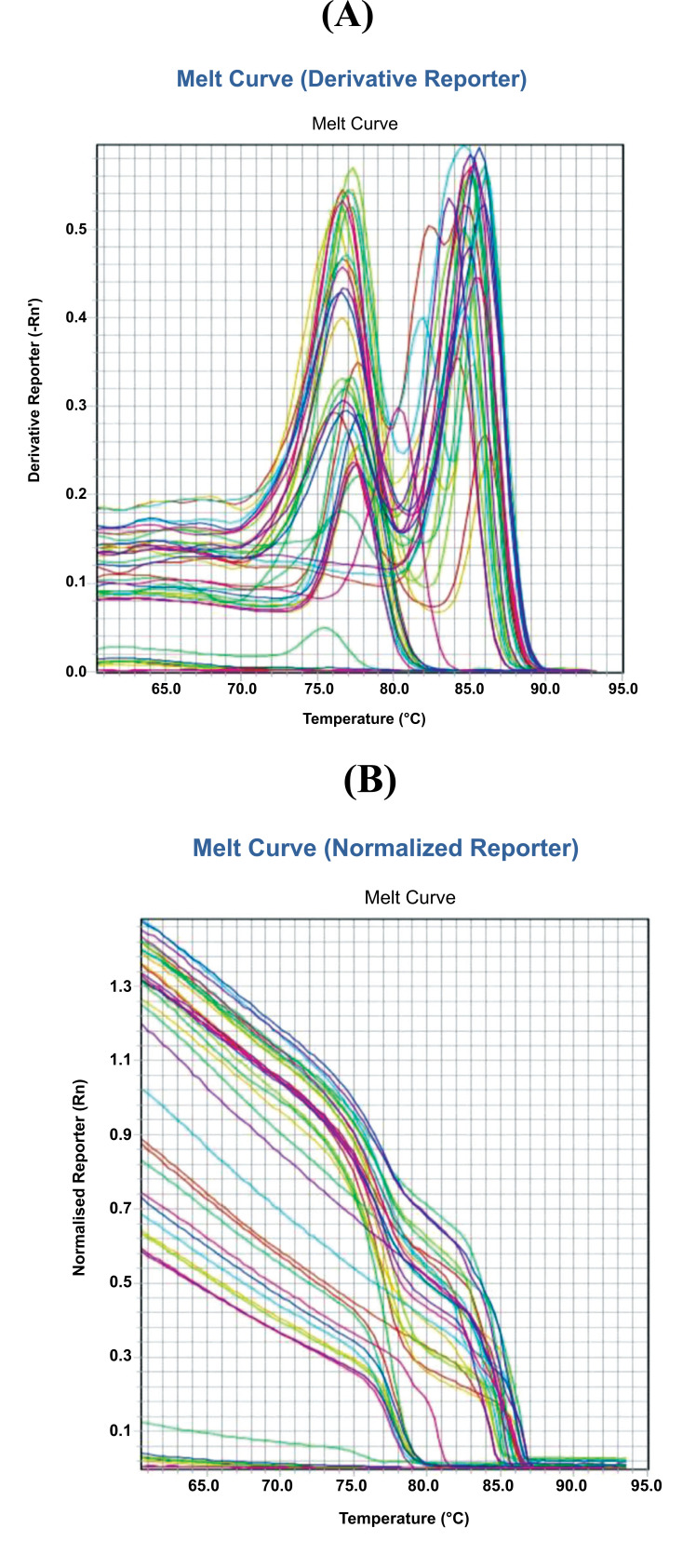
Optimization of RT PCR for p53 gene expression. (**A**). Melting curve (derivative curve); (**B**). Melting curve (normalized reporter).

**Table 1:** Comparison of the effect of filtered kretek cigarette smoke on the Sprague Dawley rat lung.

**Sprague Dawley Rat Lung**	**Treatment Groups**		-
	Group I (n=8)	Group II (n=8)	p value
- **Bronchioles:** Length (µm) Width (µm) Area (µm^2^) Perimeter (µm)	40.42 ± 1.58 14.82 ± 0.41 494.61 ± 5.62 233.87 ± 4.51	30.77 ± 0.78 9.28 ± 0.40 297.32 ± 2.53 177.84 ± 5.15	0.000 0.000 0.000 0.000
- **Respiratory bronchioles** Area (µm^2^) Perimeter (µm)	17.68 ± 0.49 26.60 ± 0.52	19.28 ± 0.38 29.28 ± 0.35	0.000 0.000
- **Expression of p53 gene**	1 ± 0.00	3.02 ± 0.69	0.000

**Abbreviations: **Group I, rats breathe using ordinary air without exposure to filtered kretek cigarette smoke. Group II, the group of rats exposed to filtered kretek cigarette smoke 1 stick/day for 3 months of treatment.

## Data Availability

All the data and supporting information is provided within the article.

## LIST OF ABBREVIATIONS

COPD: Chronic Obstructive Pulmonary Disease
RT PCR: Real-time Polymerase Chain Reaction
mRNA: Messenger ribonucleic acid
GAPDH: Glyceraldehide-3-phosphate dehydrogenase

## References

[1] Kasri R., Ahsan A., Wiyono N.H., Jacinda A., Kusuma D. (2021). New evidence of illicit cigarette consumption and government revenue loss in Indonesia.. Tob. Induc. Dis..

[2] WHO (2020). Tobacco.. https://www.who.int/news-room/fact-sheets/detail/tobacco.

[3] Jha P. (2020). The hazards of smoking and the benefits of cessation: A critical summation of the epidemiological evidence in high-income countries.. eLife.

[4] SEATCA (Southeast Asia Tobacco Control Alliance) (2018). Tobacco Control Atlas ASEAN Region.. https://seatca.org/dmdocuments/TobaccoControlAtlas_ASEANRegion_4thEd_Dec2019.pdf.

[5] Palipudi K., Mbulo L., Kosen S., Tjandra A., Kadarmanto K., Qureshi F., Andes L., Sinha D.N., Asma S. (2015). A cross sectional study of kretek smoking in Indonesia as a Major risk to public health.. Asian Pac. J. Cancer Prev..

[6] The Indonesian Sweetener and Fiber Crops Research Institute (ISFCRI) (2015). Tobacco..

[7] ITIS (Integrated Taxonomi Information System) (2020). *Syzygium aromaticum* (L.) Merr. & L.M. Perry. Taxonomic Serial No.: 506167.. https://www.itis.gov/servlet/SingleRpt/SingleRpt/.

[8] Arnez M. (2009). Tobacco and Kretek: Indonesian drugs in historical change.. https://nbn-resolving.org/urn:nbn:de:0168-ssoar-362761.

[9] Lin J., Taggart M., Borthwick L., Fisher A., Brodlie M., Sassano M.F., Tarran R., Gray M.A. (2021). Acute cigarette smoke or extract exposure rapidly activates TRPA1-mediated calcium influx in primary human airway smooth muscle cells.. Sci. Rep..

[10] Amorós-Pérez A., Cano-Casanova L., Román-Martínez M.C., Lillo-Ródenas M.Á. (2021). Comparison of particulate matter emission and soluble matter collected from combustion cigarettes and heated tobacco products using a setup designed to simulate puffing regimes.. Chem. Eng. J. Adv..

[11] Braun M., Fromm E.L., Gerber A., Klingelhöfer D., Müller R., Groneberg D.A. (2019). Particulate matter emissions of four types of one cigarette brand with and without additives: A laser spectrometric particulate matter analysis of secondhand smoke.. BMJ Open.

[12] Paul A.R., Khan F., Jain A., Saha S.C. (2021). Deposition of smoke particles in human airways with realistic waveform.. Atmosphere (Basel).

[13] González-Luis G.E., van Westering-Kroon E., Villamor-Martinez E., Huizing M.J., Kilani M.A., Kramer B.W., Villamor E. (2020). Tobacco Smoking during pregnancy is associated with increased risk of Moderate/Severe Bronchopulmonary Dysplasia: A systematic review and meta-analysis.. Front Pediatr..

[14] WHO (2019). WHO highlights huge scale of tobacco-related lung disease deaths.. https://www.who.int/news/item/29-05-2019-who-highlights-huge-scale-of-tobacco-related-lung-disease-deaths.

[15] Carvalho JL, Miranda M, Fialho AK (2020). Oral feeding with probiotic Lactobacillus rhamnosus attenuates cigarette smoke-induced COPD in C57Bl/6 mice: Relevance to inflammatory markers in human bronchial epithelial cells.. PLoS One.

[16] Shraideh Z.A., Najjar H.N. (2011). Histological changes in tissues of trachea and lung alveoli of albino rats exposed to the smoke of two types of Narghile Tobacco Products.. http://jjbs.hu.edu.jo/FILES/v4n4/Paper%20Number%205.pdf.

[17] Maina J.N., Igbokwe C.O. (2020). Comparative morphometric analysis of lungs of the semifossorial giant pouched rat (Cricetomys gambianus) and the subterranean Nigerian mole rat (*Cryptomys foxi*).. Sci. Rep..

[18] Ibe C.S., Onyeanusi B.I., Salami S.O., Nzalak J.O. (2011). Microscopic anatomy of the lower respiratory system of the African Giant Pouched Rat (*Cricetomys gambianus*, Waterhouse 1840).. Int. J. Morphol..

[19] Serra DS, De Brito KBP, Oliveira KL (2017). Respiratory system of rats exposed to pollutants arising out of heating residual glycerol.. J. Fundament. Renewable Energy Appl..

[20] Wawryk-Gawda E., Chylińska-Wrzos P., Zarobkiewicz M., Chłapek K., Jodłowska-Jędrych B. (2020). Lung histomorphological alterations in rats exposed to cigarette smoke and electronic cigarette vapour.. Exp. Ther. Med..

[21] Kim V., Criner G.J. (2013). Chronic bronchitis and chronic obstructive pulmonary disease.. Am. J. Respir. Crit. Care Med..

[22] Zuo W.L., Yang J., Gomi K., Chao I., Crystal R.G., Shaykhiev R. (2017). EGF-amphiregulin interplay in airway stem/progenitor cells links the pathogenesis of smoking-induced lesions in the human airway epithelium.. Stem Cells.

[23] Chauhan N.S., Sood D., Takkar P., Dhadwal D.S., Kapila R. (2019). Quantitative assessment of airway and parenchymal components of chronic obstructive pulmonary disease using thin-section helical computed tomography.. Pol. J. Radiol..

[24] Fischer M. (2017). Census and evaluation of p53 target genes.. Oncogene.

[25] Petre C.E., Sin S.H., Dittmer D.P. (2007). Functional p53 signaling in Kaposi’s sarcoma-associated herpesvirus lymphomas: Implications for therapy.. J. Virol..

[26] Livak K.J., Schmittgen T.D. (2001). Analysis of relative gene expression data using real-time quantitative PCR and the 2(-Delta Delta C(T)) Method.. Methods.

[27] Sarıkaya Y., Kahya N.D., Pekdemir A.D., Önal M. (2020). Removing tar and nicotine from mainstream cigarette smoke using sepiolite-modified filter tips.. Clay Miner..

[28] Wallin C., Sholts S.B., Österlund N., Luo J., Jarvet J., Roos P.M., Ilag L., Gräslund A., Wärmländer S.K.T.S. (2017). Alzheimer’s disease and cigarette smoke components: Effects of nicotine, PAHs, and Cd(II), Cr(III), Pb(II), Pb(IV) ions on amyloid-β peptide aggregation.. Sci. Rep..

[29] Anggraeni N.K.N., Djoko D.J.H.S., Juswono U.P. (2020). Identification of free radical in main stream cigarette smoke in cigarette with clove mix (Kretek) and cigarette without clove mix (White).. Buletin Fisika.

[30] Strzelak A., Ratajczak A., Adamiec A., Feleszko W. (2018). Tobacco smoke induces and alters immune responses in the lung triggering inflammation, allergy, asthma and other lung diseases: A mechanistic review.. Int. J. Environ. Res. Public Health.

[31] Laborada J., Cohen P.R. (2021). Smoker’s mustache revisited: Upper lip hair yellow discoloration associated with tobacco.. Cureus.

[32] Nadhiroh S.R., Djokosujono K., Utari D.M., Hasugian A.R. (2020). Questionnaire-based environmental tobacco smoke exposure and hair nicotine levels in 6-month-old infants: A validation study in Indonesia.. Glob. Pediatr. Health.

[33] Murphy S.E. (2021). Biochemistry of nicotine metabolism and its relevance to lung cancer.. J. Biol. Chem..

[34] Inukai T., Kaji S., Kataoka H. (2018). Analysis of nicotine and cotinine in hair by on-line in-tube solid-phase microextraction coupled with liquid chromatography-tandem mass spectrometry as biomarkers of exposure to tobacco smoke.. J. Pharm. Biomed. Anal..

[35] Trüeb R.M. (2015). The impact of oxidative stress on hair.. Int. J. Cosmet. Sci..

[36] Kim T., Kang J. (2021). Association between dual use of e-cigarette and cigarette and chronic obstructive pulmonary disease: An analysis of a nationwide representative sample from 2013 to 2018.. BMC Pulm. Med..

[37] Tjahyadi D, Parwanto E (2023). Effects of low-dose filtered kretek cigarette smoke on bronchial smooth muscle in male Sprague-Dawley rats.. Univ. Med..

[38] Tjahyadi D., Parwanto E., Amalia H., Digambiro R.A., Edy H.J., Oladimeji A.V. (2023). Decreased density of pyramidal cells in the cerebral cortex, and Purkinje cells in the cerebellar cortex of Sprague-Dawley rats after being exposed to filtered kretek cigarette smoke.. J. Bio. Res..

[39] Burgel P-R., Martin, Frija (2013). Dysfunctional lung anatomy and small airways degeneration in COPD.. Int. J. Chron. Obstruct. Pulmon. Dis..

[40] Chookliang A., Sricharoenvej S., Lanlua P. (2019). Alterations in the bronchioles of rat lungs with chronic diabetes.. http://conf.ejikei.org/ICTSS/2019/proceedings/materials/proc_files/GS/ICTSS2019_GS_A007/CameraReady_ICTSS2019_GS_A007_Ms.Chookliang.pdf.

[41] Yanko R., Levashov M., Chaka E., Safonov S. (2021). Morphofunctional state of the lungs respiratory part in normotensive and hypertensive rats after combined exposure to intermittent hypoxia and melatonin.. J. Educ. Health Sport.

[42] Whitsett J.A. (2018). Airway epithelial differentiation and mucociliary clearance.. Ann. Am. Thorac. Soc..

[43] Zhang H., Yu W., Ji L., Zhong Y., Lin Y., Ying H., Yu C., Li C. (2021). Guifu dihuang pills ameliorated mucus hypersecretion by suppressing Muc5ac expression and inactivating the ERK-SP1 pathway in lipopolysaccharide/cigarette smoke-induced mice.. Evid. Based Complement. Alternat. Med..

[44] Duan J., Cheng W., Zeng Y., Chen Y., Cai S., Li X., Zhu Y., Chen M., Zhou M., Ma L., Liu Q., Chen P. (2020). Characteristics of Patients with chronic obstructive pulmonary disease exposed to different environmental risk factors: A large cross-sectional study.. Int. J. Chron. Obstruct. Pulmon. Dis..

[45] Safitri W., Martini S., Artanti K.D., Li C.Y. (2021). Smoking from a younger age is the dominant factor in the incidence of chronic obstructive pulmonary disease: Case-control study.. Int. J. Environ. Res. Public Health.

[46] Seton-Rogers S. (2020). Complex effects of tobacco on lung tissue.. Nat. Rev. Cancer.

[47] Seiler C.L., Song J.M., Kotandeniya D., Chen J., Kono T.J.Y., Han Q., Colwell M., Auch B., Sarver A.L., Upadhyaya P., Ren Y., Faulk C., De Flora S., La Maestra S., Chen Y., Kassie F., Tretyakova N.Y. (2020). Inhalation exposure to cigarette smoke and inflammatory agents induces epigenetic changes in the lung.. Sci. Rep..

[48] Aredo J.V., Luo S.J., Gardner R.M., Sanyal N., Choi E., Hickey T.P., Riley T.L., Huang W.Y., Kurian A.W., Leung A.N., Wilkens L.R., Robbins H.A., Riboli E., Kaaks R., Tjønneland A., Vermeulen R.C.H., Panico S., Le Marchand L., Amos C.I., Hung R.J., Freedman N.D., Johansson M., Cheng I., Wakelee H.A., Han S.S. (2021). Tobacco smoking and risk of second primary lung cancer.. J. Thorac. Oncol..

[49] Vinay S., Dharmashekara C., Prasad A. (2021). Smoking carcinogens and lung cancer-a review.. Asian J. Pharm. Clin. Res..

[50] Nuryunarsih D., Lewis S., Langley T. (2021). Health risks of Kretek Cigarettes: A systematic review.. Nicotine Tob. Res..

[51] Sadia H., Ahmad Bhinder M., Irshad A., Zahid B., Ahmed R., Ashiq S., Malik K., Riaz M., Nadeem T., Ashiq K., Akbar A. (2020). Determination of expression profile of p53 gene in different grades of breast cancer tissues by real time PCR.. Afr. Health Sci..

[52] Shi D., Jiang P. (2021). A different facet of p53 Function: Regulation of immunity and inflammation during tumor development.. Front. Cell Dev. Biol..

[53] Singh R., Patel K., Patel J., Patel P. (2023). Association of interactions between Metabolic ‘Caretaker’ Genes, p53, MDM2, and Tobacco Use with the Risk of Oral Cancer: A multifactor dimensionality reduction approach.. Asian Pac. J. Cancer Prev..

[54] Parwanto M.L.E., Wratsangka R., Guyansyah A., Anggraeni K. (2019). Mutation of the Fas-promoter-670 gene, AA to GA in the normal cervix-epithelial-cells of high risk Indonesian mother: A case report.. Bali Med. J..

[55] Parwanto E., Wratsangka R., Guyansyah A., Anggraeni K., Digambiro R.A., Tjahyadi D., Arkeman H., Widyatama H.G., Edy H.J., Edy Y.J. (2021). The change of cell biometric and its nucleus on cervical-squamous-epithelial-cell with GA genotype of Fas-promoter-670 gene, high-risk human papillomavirus and *Candida* species infection: A case report.. Bali Med. J..

